# Supplementary material for: Lipid Profile Characterization and Lipoprotein Comparison of Extracellular Vesicles from Human Plasma and Serum
Source: Metabolites. 2019 Nov 1;9(11):259. doi: 10.3390/metabo9110259 (PMC6918450; doi:10.3390/metabo9110259)
Supplement: Supplementary file 1 [file metabolites-09-00259-s001.zip › EV lipidomics sup-figures metabolites_191101US_revfin.pptx]

## Slide 1
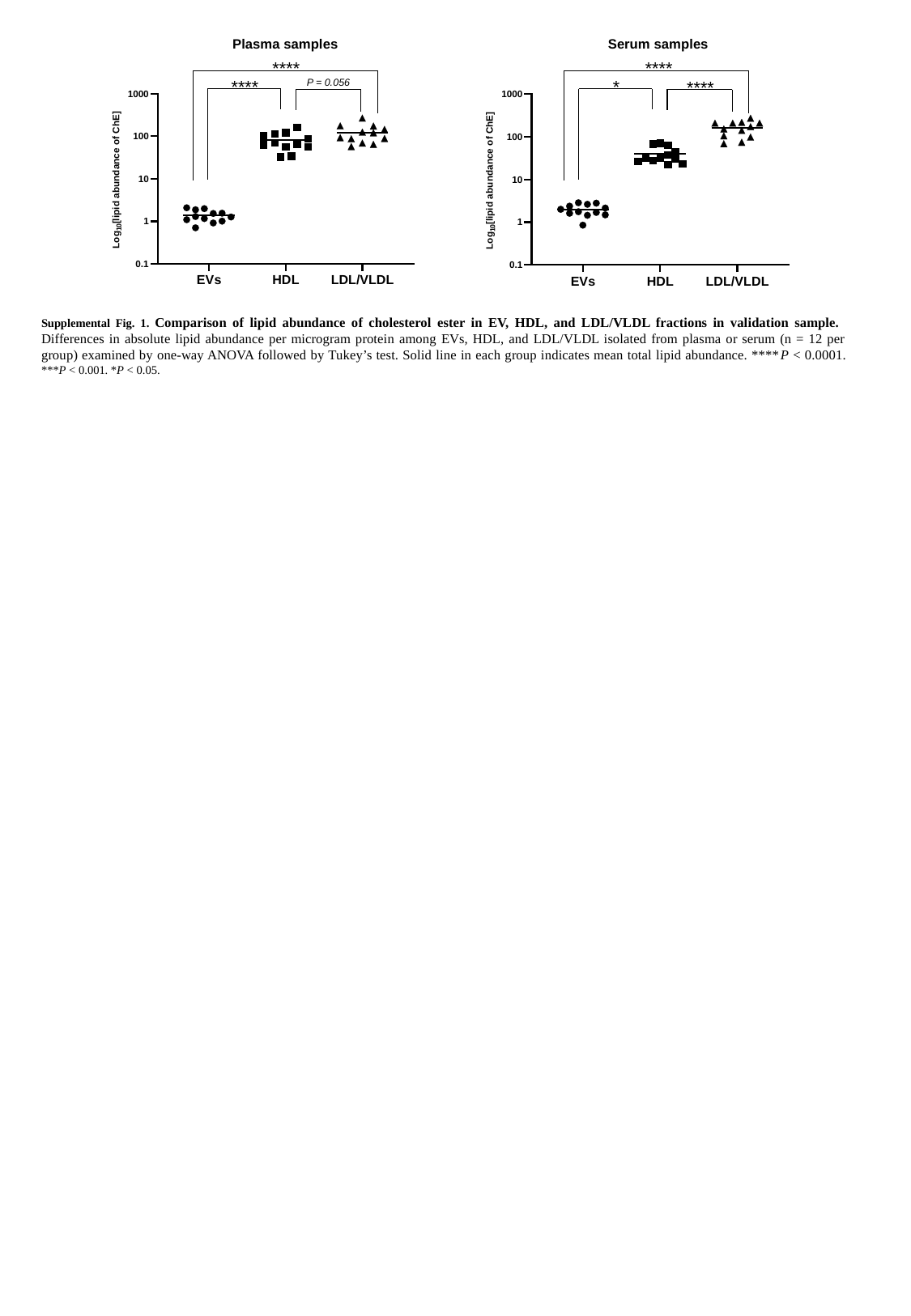

Plasma samples
Serum samples
****
****
****
*
P = 0.056
****
Supplemental Fig. 1. Comparison of lipid abundance of cholesterol ester in EV, HDL, and LDL/VLDL fractions in validation sample. Differences in absolute lipid abundance per microgram protein among EVs, HDL, and LDL/VLDL isolated from plasma or serum (n = 12 per group) examined by one-way ANOVA followed by Tukey’s test. Solid line in each group indicates mean total lipid abundance. ****P < 0.0001. ***P < 0.001. *P < 0.05.

## Slide 2
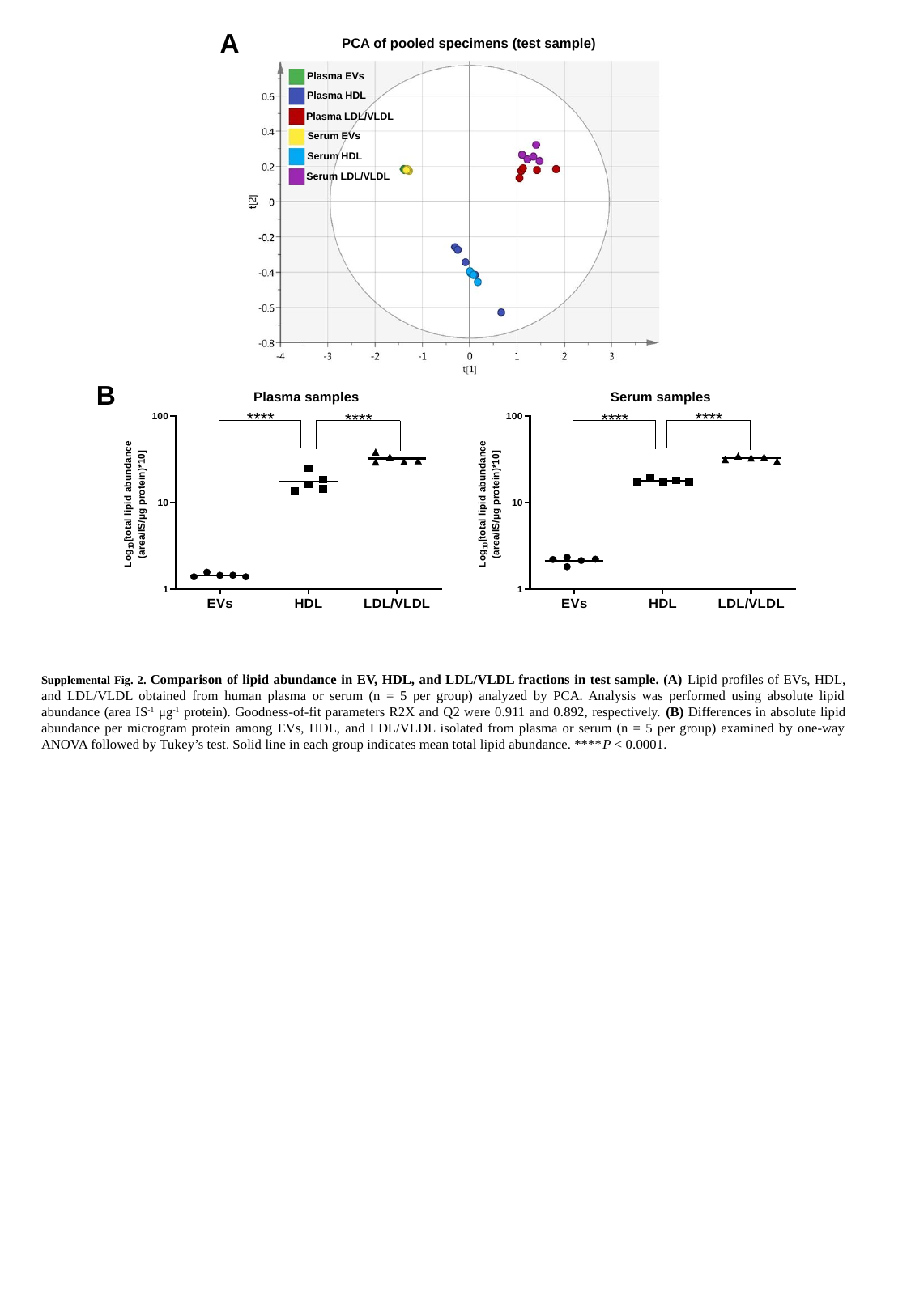

A
PCA of pooled specimens (test sample)
Plasma EVs
Plasma HDL
Plasma LDL/VLDL
Serum EVs
Serum HDL
Serum LDL/VLDL
B
Plasma samples
Serum samples
****
****
****
****
Supplemental Fig. 2. Comparison of lipid abundance in EV, HDL, and LDL/VLDL fractions in test sample. (A) Lipid profiles of EVs, HDL, and LDL/VLDL obtained from human plasma or serum (n = 5 per group) analyzed by PCA. Analysis was performed using absolute lipid abundance (area IS-1 μg-1 protein). Goodness-of-fit parameters R2X and Q2 were 0.911 and 0.892, respectively. (B) Differences in absolute lipid abundance per microgram protein among EVs, HDL, and LDL/VLDL isolated from plasma or serum (n = 5 per group) examined by one-way ANOVA followed by Tukey’s test. Solid line in each group indicates mean total lipid abundance. ****P < 0.0001.

## Slide 3
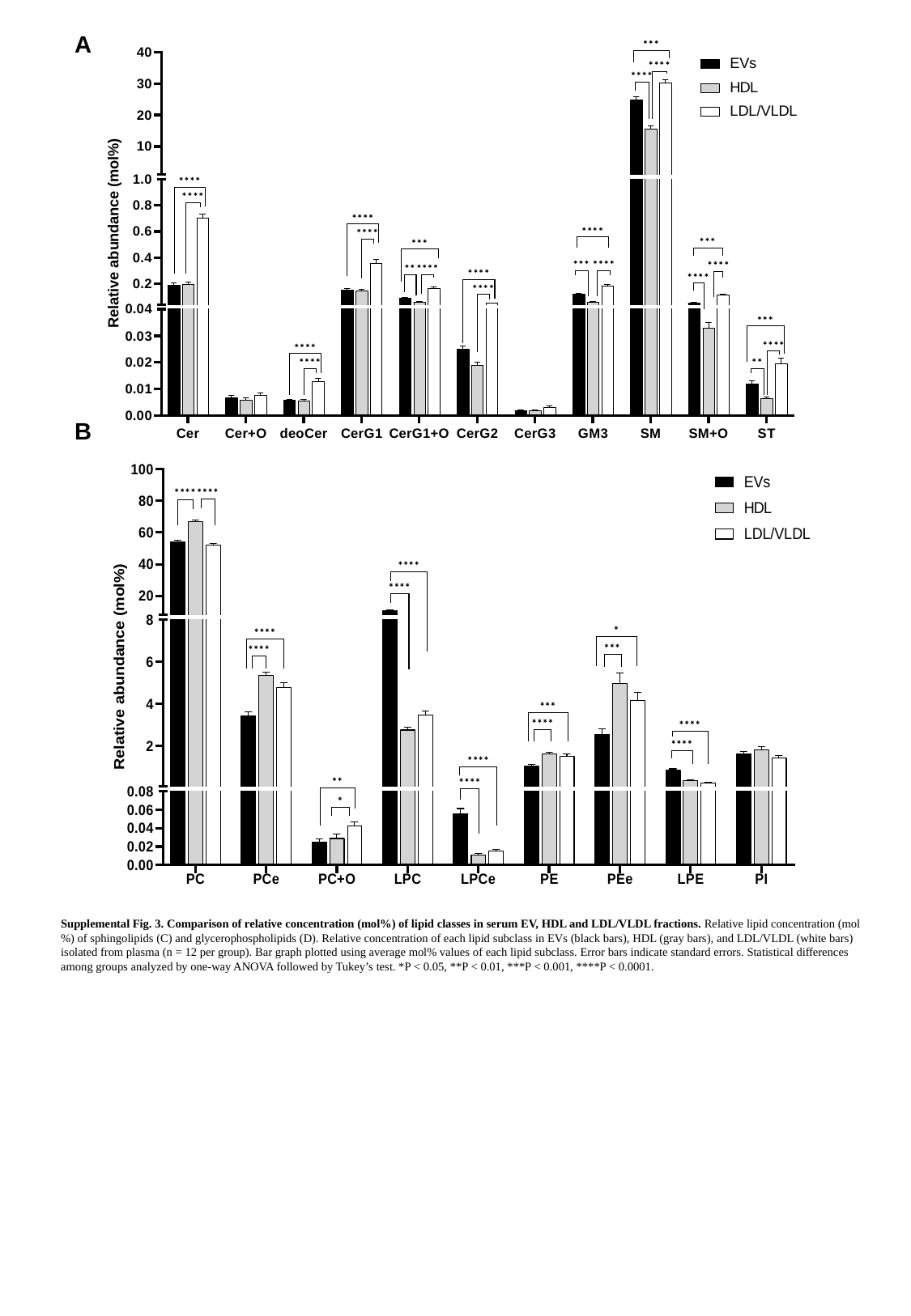

A
B
Supplemental Fig. 3. Comparison of relative concentration (mol%) of lipid classes in serum EV, HDL and LDL/VLDL fractions. Relative lipid concentration (mol%) of sphingolipids (C) and glycerophospholipids (D). Relative concentration of each lipid subclass in EVs (black bars), HDL (gray bars), and LDL/VLDL (white bars) isolated from plasma (n = 12 per group). Bar graph plotted using average mol% values of each lipid subclass. Error bars indicate standard errors. Statistical differences among groups analyzed by one-way ANOVA followed by Tukey’s test. *P < 0.05, **P < 0.01, ***P < 0.001, ****P < 0.0001.

## Slide 4
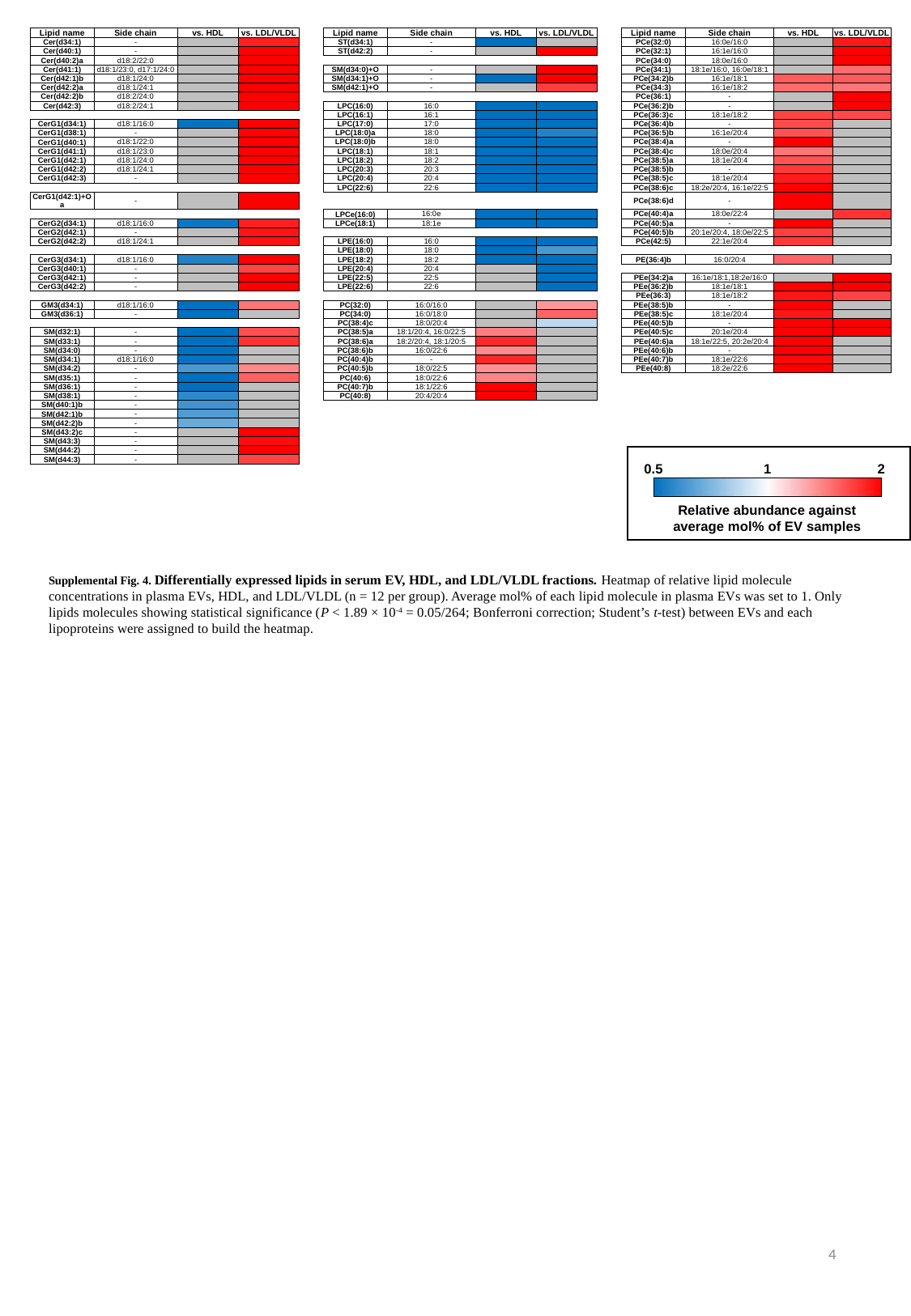

| Lipid name | Side chain | vs. HDL | vs. LDL/VLDL | | | Lipid name | Side chain | vs. HDL | vs. LDL/VLDL | | | Lipid name | Side chain | vs. HDL | vs. LDL/VLDL |
| --- | --- | --- | --- | --- | --- | --- | --- | --- | --- | --- | --- | --- | --- | --- | --- |
| Cer(d34:1) | - | | | | | ST(d34:1) | - | | | | | PCe(32:0) | 16:0e/16:0 | | |
| Cer(d40:1) | - | | | | | ST(d42:2) | - | | | | | PCe(32:1) | 16:1e/16:0 | | |
| Cer(d40:2)a | d18:2/22:0 | | | | | | | | | | | PCe(34:0) | 18:0e/16:0 | | |
| Cer(d41:1) | d18:1/23:0, d17:1/24:0 | | | | | SM(d34:0)+O | - | | | | | PCe(34:1) | 18:1e/16:0, 16:0e/18:1 | | |
| Cer(d42:1)b | d18:1/24:0 | | | | | SM(d34:1)+O | - | | | | | PCe(34:2)b | 16:1e/18:1 | | |
| Cer(d42:2)a | d18:1/24:1 | | | | | SM(d42:1)+O | - | | | | | PCe(34:3) | 16:1e/18:2 | | |
| Cer(d42:2)b | d18:2/24:0 | | | | | | | | | | | PCe(36:1) | - | | |
| Cer(d42:3) | d18:2/24:1 | | | | | LPC(16:0) | 16:0 | | | | | PCe(36:2)b | - | | |
| | | | | | | LPC(16:1) | 16:1 | | | | | PCe(36:3)c | 18:1e/18:2 | | |
| CerG1(d34:1) | d18:1/16:0 | | | | | LPC(17:0) | 17:0 | | | | | PCe(36:4)b | - | | |
| CerG1(d38:1) | - | | | | | LPC(18:0)a | 18:0 | | | | | PCe(36:5)b | 16:1e/20:4 | | |
| CerG1(d40:1) | d18:1/22:0 | | | | | LPC(18:0)b | 18:0 | | | | | PCe(38:4)a | - | | |
| CerG1(d41:1) | d18:1/23:0 | | | | | LPC(18:1) | 18:1 | | | | | PCe(38:4)c | 18:0e/20:4 | | |
| CerG1(d42:1) | d18:1/24:0 | | | | | LPC(18:2) | 18:2 | | | | | PCe(38:5)a | 18:1e/20:4 | | |
| CerG1(d42:2) | d18:1/24:1 | | | | | LPC(20:3) | 20:3 | | | | | PCe(38:5)b | - | | |
| CerG1(d42:3) | - | | | | | LPC(20:4) | 20:4 | | | | | PCe(38:5)c | 18:1e/20:4 | | |
| | | | | | | LPC(22:6) | 22:6 | | | | | PCe(38:6)c | 18:2e/20:4, 16:1e/22:5 | | |
| CerG1(d42:1)+Oa | - | | | | | | | | | | | PCe(38:6)d | - | | |
| | | | | | | LPCe(16:0) | 16:0e | | | | | PCe(40:4)a | 18:0e/22:4 | | |
| CerG2(d34:1) | d18:1/16:0 | | | | | LPCe(18:1) | 18:1e | | | | | PCe(40:5)a | - | | |
| CerG2(d42:1) | - | | | | | | | | | | | PCe(40:5)b | 20:1e/20:4, 18:0e/22:5 | | |
| CerG2(d42:2) | d18:1/24:1 | | | | | LPE(16:0) | 16:0 | | | | | PCe(42:5) | 22:1e/20:4 | | |
| | | | | | | LPE(18:0) | 18:0 | | | | | | | | |
| CerG3(d34:1) | d18:1/16:0 | | | | | LPE(18:2) | 18:2 | | | | | PE(36:4)b | 16:0/20:4 | | |
| CerG3(d40:1) | - | | | | | LPE(20:4) | 20:4 | | | | | | | | |
| CerG3(d42:1) | - | | | | | LPE(22:5) | 22:5 | | | | | PEe(34:2)a | 16:1e/18:1,18:2e/16:0 | | |
| CerG3(d42:2) | - | | | | | LPE(22:6) | 22:6 | | | | | PEe(36:2)b | 18:1e/18:1 | | |
| | | | | | | | | | | | | PEe(36:3) | 18:1e/18:2 | | |
| GM3(d34:1) | d18:1/16:0 | | | | | PC(32:0) | 16:0/16:0 | | | | | PEe(38:5)b | - | | |
| GM3(d36:1) | - | | | | | PC(34:0) | 16:0/18:0 | | | | | PEe(38:5)c | 18:1e/20:4 | | |
| | | | | | | PC(38:4)c | 18:0/20:4 | | | | | PEe(40:5)b | - | | |
| SM(d32:1) | - | | | | | PC(38:5)a | 18:1/20:4, 16:0/22:5 | | | | | PEe(40:5)c | 20:1e/20:4 | | |
| SM(d33:1) | - | | | | | PC(38:6)a | 18:2/20:4, 18:1/20:5 | | | | | PEe(40:6)a | 18:1e/22:5, 20:2e/20:4 | | |
| SM(d34:0) | - | | | | | PC(38:6)b | 16:0/22:6 | | | | | PEe(40:6)b | - | | |
| SM(d34:1) | d18:1/16:0 | | | | | PC(40:4)b | - | | | | | PEe(40:7)b | 18:1e/22:6 | | |
| SM(d34:2) | - | | | | | PC(40:5)b | 18:0/22:5 | | | | | PEe(40:8) | 18:2e/22:6 | | |
| SM(d35:1) | - | | | | | PC(40:6) | 18:0/22:6 | | | | | | | | |
| SM(d36:1) | - | | | | | PC(40:7)b | 18:1/22:6 | | | | | | | | |
| SM(d38:1) | - | | | | | PC(40:8) | 20:4/20:4 | | | | | | | | |
| SM(d40:1)b | - | | | | | | | | | | | | | | |
| SM(d42:1)b | - | | | | | | | | | | | | | | |
| SM(d42:2)b | - | | | | | | | | | | | | | | |
| SM(d43:2)c | - | | | | | | | | | | | | | | |
| SM(d43:3) | - | | | | | | | | | | | | | | |
| SM(d44:2) | - | | | | | | | | | | | | | | |
| SM(d44:3) | - | | | | | | | | | | | | | | |
0.5
1
2
Relative abundance against
average mol% of EV samples
Supplemental Fig. 4. Differentially expressed lipids in serum EV, HDL, and LDL/VLDL fractions. Heatmap of relative lipid molecule concentrations in plasma EVs, HDL, and LDL/VLDL (n = 12 per group). Average mol% of each lipid molecule in plasma EVs was set to 1. Only lipids molecules showing statistical significance (P < 1.89 × 10-4 = 0.05/264; Bonferroni correction; Student’s t-test) between EVs and each lipoproteins were assigned to build the heatmap.
4

## Slide 5
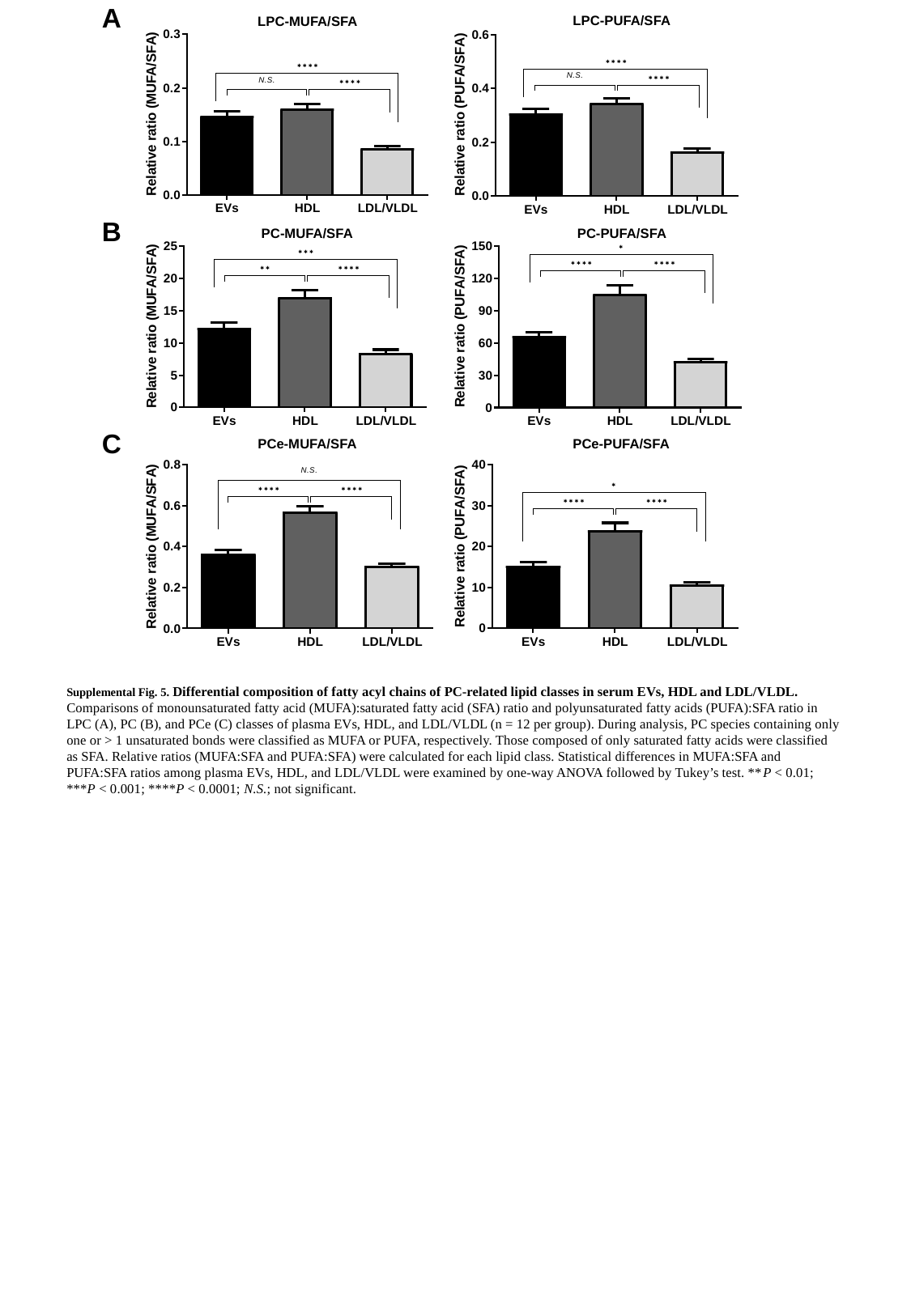

A
LPC-PUFA/SFA
LPC-MUFA/SFA
B
PC-MUFA/SFA
PC-PUFA/SFA
C
PCe-MUFA/SFA
PCe-PUFA/SFA
Supplemental Fig. 5. Differential composition of fatty acyl chains of PC-related lipid classes in serum EVs, HDL and LDL/VLDL. Comparisons of monounsaturated fatty acid (MUFA):saturated fatty acid (SFA) ratio and polyunsaturated fatty acids (PUFA):SFA ratio in LPC (A), PC (B), and PCe (C) classes of plasma EVs, HDL, and LDL/VLDL (n = 12 per group). During analysis, PC species containing only one or > 1 unsaturated bonds were classified as MUFA or PUFA, respectively. Those composed of only saturated fatty acids were classified as SFA. Relative ratios (MUFA:SFA and PUFA:SFA) were calculated for each lipid class. Statistical differences in MUFA:SFA and PUFA:SFA ratios among plasma EVs, HDL, and LDL/VLDL were examined by one-way ANOVA followed by Tukey’s test. **P < 0.01; ***P < 0.001; ****P < 0.0001; N.S.; not significant.
